# Atmospheric Sulfuric Acid Dimer Formation in a Polluted Environment

**DOI:** 10.3390/ijerph19116848

**Published:** 2022-06-03

**Authors:** Ke Yin, Shixin Mai, Jun Zhao

**Affiliations:** 1 School of Atmospheric Sciences, Guangdong Province Key Laboratory for Climate Change and Natural Disaster Studies, and Southern Marine Science and Engineering Guangdong Laboratory (Zhuhai), Sun Yat-sen University, Zhuhai 519082, China; yink6@mail2.sysu.edu.cn (K.Y.); maishx3@mail2.sysu.edu.cn (S.M.); 2 Guangdong Provincial Observation and Research Station for Climate Environment and Air Quality Change in the Pearl River Estuary, Zhuhai 519082, China; 3 Key Laboratory of Tropical Atmosphere-Ocean System, Ministry of Education, Zhuhai 519082, China

**Keywords:** atmospheric nucleation, sulfuric acid dimer, new particle formation, sulfur-rich environments, ion-induced clustering, amines

## Abstract

New particle formation (NPF) contributes significantly to atmospheric particle number concentrations and cloud condensation nuclei (CCN). In sulfur-rich environments, field measurements have shown that sulfuric acid dimer formation is likely the critical step in NPF. We investigated the dimer formation process based upon the measured sulfuric acid monomer and dimer concentrations, along with previously reported amine concentrations in a sulfur-rich atmosphere (Atlanta, USA). The average sulfuric acid concentration was in the range of 1.7 × 10^7^–1.4 × 10^8^ cm^−3^ and the corresponding neutral dimer concentrations were 4.1 × 10^5^–5.0 × 10^6^ cm^−3^ and 2.6 × 10^5^–2.7 × 10^6^ cm^−3^ after sub-collision and collision ion-induced clustering (IIC) corrections, respectively. Two previously proposed acid–base mechanisms (namely *AA* and *AB*) were employed to respectively estimate the evaporation rates of the dimers and the acid–amine complexes. The results show evaporation rates of 0.1–1.3 s^−1^ for the dimers based on the simultaneously measured average concentrations of the total amines, much higher than those (1.2–13.1 s^−1^) for the acid–amine complexes. This indicates that the mechanism for dimer formation is likely *AA* through the formation of more volatile dimers in the initial step of the cluster formation.

## 1. Introduction

Atmospheric particulate matter (PM) contributes significantly to regional and local air pollution, and adversely affects human health. The sizes of atmospheric particles can range from nano- to micrometers, notably from the molecular nucleating cluster scale (~1–2 nm), PM_2.5_ (less than 2.5 μm), PM_10_ (less than 10 μm), up to 100 μm. The atmospheric PM has various sources, including primary and secondary origins, among which new particle formation (NPF), a frequently observed atmospheric phenomenon, is an important contributor to the total number and loading of atmospheric PM. The newly formed particles can grow via several processes, including condensation as well as coagulation to larger sizes (above ~50 nm), which can then act as cloud condensation nuclei (CCN), affecting cloud formation and the atmospheric radiation budget. 

To date, field measurements and laboratory experiments have shown that many atmospheric species can serve as precursors of NPF and early growth participants, including sulfuric acid (with water), ammonia, organic acids, amines, etc. [1,2,3,4]. Gas-phase sulfuric acid is commonly believed to be one of the earliest and most important species for atmospheric nucleation and early growth processes, and early field measurements have found strong correlations between sulfuric acid concentrations and the production rates of newly formed nanoparticles [5]. These correlations may imply the number of sulfuric acid molecules contained in the nucleating clusters (~1–2 nm) that determine the nucleation rate, which can vary from one to four under various environmental conditions [6,7,8,9,10]. Atmospheric nucleation involving sulfuric acid and organic acids was pioneered by Zhang and co-workers [1,11]. Many laboratory and theoretical studies have been carried out to investigate sulfuric acid–organic acid nucleation [12,13,14,15]. Recent field measurements showed that highly oxidized molecules (HOMs) may play dominant roles in the early stage of the production of nanoparticles, especially in sulfur-poor environments [16,17,18,19,20,21]. Organic enhanced cluster formation was recently summarized in a review article [22]. However, the detailed nucleation mechanism involving organics still remains poorly understood.

In a sulfur-rich atmosphere, gaseous sulfuric acid plays a major role in the boundary layer NPF via the formation of neutral molecular clusters, although ion-mediated cluster formation would be significant in the upper free atmosphere. Atmospheric neutral sulfuric acid clusters were detected and quantified using chemical ionization mass spectrometric techniques [9,23]; for example, the Cluster CIMS originally developed at the National Center for Atmospheric Research (NCAR, USA) and the CI-APi-TOF developed jointly by Aerodyne Research (USA) and Tofwerf AG (Switzerland). The latter has been widely applied in field and laboratory measurements, including the cosmics leaving outdoor droplets (CLOUD) chamber experiments. The Cluster CIMS can measure atmospheric neutral sulfuric acid dimers (up to tetramers) in various environments, including urban/suburban, remote, and coastal areas [9,24]. The measurements of nucleating molecular clusters bridge the gap between vapor molecules (about several tenths of a nanometer) and newly formed nanoparticles (about one to several nanometers) during NPF events [25]. A recent review article summarizes measurements of atmospheric nanoparticles, including those for the molecular clusters [26].

Currently, the governing mechanisms for atmospheric nucleation under sulfur-rich environments are not fully understood. However, both field and laboratory measurements clearly demonstrate the critical roles of neutral sulfuric acid dimer formation during NPF. Two mechanisms have been proposed for dimer formation based on the acid–base theory, and are tentatively denoted as *AA* and *AB* in this paper. Combining environmental chamber experiments and field measurements in sulfur-rich environments (i.e., Atlanta and Mexico City), Chen et al. [10] proposed a conceptual acid–base chemical reaction model for estimates of nucleation rates (*AA*). In the *AA* model, more volatile (*MV*) dimers are initially formed, and subsequently stabilized by a base molecule, leading to less volatile (*LV*) dimers almost without evaporation, and clusters larger than trimers that grow spontaneously. This model explains, very well, atmospheric nucleation, and predicts, reasonably well, the nucleation rates in sulfur-rich polluted environments [10]. A close-to-collision-limited sulfuric acid dimer formation was also observed in a laboratory study [27] using a laminar flow tube reactor, further demonstrating the crucial steps for dimer formation and the base stabilization effects during NPF.

Alternatively, another acid–base model (*AB*) was proposed via the initial formation of a sulfuric acid–base complex, subsequently stabilized by a sulfuric acid molecule or the complex itself. The base compounds are ubiquitous in ambient air, including ammonia and amines, and the latter were found to enhance atmospheric nucleation more effectively than the former in theoretical and laboratory studies [28,29,30,31,32,33,34,35,36]. Among many amine species, dimethylamine (DMA) is the most abundant amine compound in the atmosphere [37,38]. A H_2_SO_4_–DMA mechanism was proposed to interpret the observed high concentrations of sulfuric acid dimers during NPF events in Shanghai [39], consistent with the H_2_SO_4_–DMA nucleation from the CLOUD experiments. A hybrid *AB* mechanism, H_2_SO_4_–DMA–NH_3_, was suggested for the nucleation process under high concentrations of ammonia, according to a modeling study based on field measurement data [40]. Nevertheless, this H_2_SO_4_–DMA mechanism can also occur in the open ocean and the coastal atmosphere around the Antarctic Peninsula, where air masses originate from the nearby ice-covered sea and its marginal ice zone [41]. 

In urban Beijing, the sulfuric acid–amine (*AB*) mechanism was also considered as the dominant pathway for NPF base on long-term atmospheric measurements [42,43,44], especially under high condensational sinks. The condensation of H_2_SO_4_ and its amine clusters contributes more significantly to the growth of particles less than 3 nm, compared to those above 3 nm [45]. However, the sub-3 nm particles grew rather slowly due to the high level of background scavenging aerosols [46]. In addition, oxidized organics were also involved in early growth once the fast clustering between sulfuric acid and amine molecules was initiated during NPF events, which enables the growth of particles toward climate- and health-impacting sizes [47]. 

In addition to ammonia/amine and organic acids, inorganic acids, such as nitric acid, can enhance H_2_SO_4_–base clusters. For example, it could greatly enhance sulfuric acid–ammonia nucleation in the cold upper free troposphere or in an extremely low temperature boundary layer [48,49]. A recent modeling study showed that nitric acid can enhance sulfuric acid–dimethylamine nucleation in the polluted boundary layer, such as in Beijing [48]. The enhancement factor could be as high as 80 for the formation rates, and more than 20-fold for the number concentrations of nucleation clusters in polluted regions, such as Beijing. The stability of the acid–base clusters was evaluated, and gas-phase acidity was found to be most critical among many factors (aqueous-phase acidity, heterodimer proton transference, vapor pressure, dipole moment and polarizability, etc.), using a predictive model [50]. 

This study reported field measurements of sulfuric acid dimers during NPF events in Atlanta (USA), a polluted sulfur-rich urban environment. Sulfuric acid and its molecular clusters were measured using the NCAR’s Cluster CIMS. The formation mechanisms (*AA* vs. *AB*) of sulfuric acid dimers were investigated through constraining the simultaneously measured concentrations of amines reported previously [51]. 

## 2. Materials and Methods

The measurements were performed at the Jefferson Street (JST) site in Atlanta from mid-July through August 2009. The site is regularly impacted by plumes from several nearby coal-fired power plants during the campaign. Detailed information on the surrounding characteristics of the site can be found in the SI of [10]. Gas-phase sulfuric acid and its nucleating molecular clusters were measured using the NCAR Cluster CIMS. Detailed description of the Cluster CIMS can be found elsewhere [9], and only a brief description is given here. The instrument employed nitric acid (HNO_3_) as a reagent to generate the primary ions NO_3_^−^▪(HNO_3_)_n_ (n = 1, 2) by introducing a small pure N_2_ flow (usually several cubic centimeters per minute) into a temperature-regulated vial containing HNO_3_, and subsequently joining the main carrier gas flow (about 1–2 lpm pure N_2_ flow). The combined flow is then passed through a radioactive ^241^Am ion source. Sulfuric acid and its molecular clusters react with the primary ions to produce the corresponding ions/cluster ions, which are then transported and extracted through a small aperture (~100 μm) into the differentially pumped vacuum chamber. The ions/cluster ions were guided through a conical octopole, and mass analyzed by a quadrupole mass spectrometer. The Cluster CIMS was calibrated using an electrospray coupled to a high-resolution differential mobility analyzer (ES-HDMA) [9], and the resultant calibration factors were used when calculating the concentrations of the measured compounds/clusters. In addition to the Cluster CIMS, several other advanced instruments were employed simultaneously; particle number and size distribution was measured by a scanning mobility particle spectrometer (SMPS) [52], and concentrations of basic gaseous compounds (ammonia and amines) by ambient-pressure proton transfer mass spectrometry (AmPMS). The SMPS measurements provide the temporal distribution of particle size and concentration, which can identify NPF events. The concentrations of amines were taken from Hanson et al. [51]. Since sulfuric acid monomers and dimers are short-lived, here we report their concentrations representative of the measurement site.

Since the Cluster CIMS uses a quadrupole mass analyzer, a typical unit mass resolution is achieved for such an analyzer, and hence background corrections were needed when calculating the concentrations of sulfuric acid monomers and dimers. The interferences of background signals might come from various sources, including variations in ambient temperature and photochemical oxidation species during the day. The backgrounds of sulfuric acid monomers and dimers were observed to be low at night and in the early morning. Here, we use *m/z* 166, likely corresponding to malonic acid from photochemical formation, as a reference for background correction (*BGC*). The chosen *m/z* 166 as a reference compound is reasonable since the background variations for both sulfuric acid monomers and dimers likely follow a similar trend as the *m/z* 166. We performed a lognormal curve fit through the normalized (to the primary ion signal at *m/z* 125) signals of *m/z* 166, and assumed that the backgrounds for sulfuric acid and its cluster ions followed a similar temporal trend [10]. The lognormal function can provide a reasonable fit to the data, and is given by:

(1) $$BGC=SF*\left\{y_{0}+A\cdot exp\left[-{\left(\frac{ln\left(\frac{x}{x_{0}}\right)}{width}\right)}^{2}\right]\right\}\;$$

where the fitting parameters (*y*_0_, *A*, and *x*_0_) and the scaling factor *SF* varied daily, and separated fits were performed for each NPF of interest in this study. The lognormal function fit was applied to *m/z* 166, given that the variation in its signal during the day was smooth, which was the case for most of the events; however, for some events, i.e., those on 6, 7, 10, and 12 August, special care was needed for the *BGC*s, as detailed in the Supplementary Material (Table S1 and Figures S1–S8). Sulfuric acid monomer and dimer signals were then obtained by subtracting the above *BGC*s from measurements.

Here, we correlate sulfuric acid dimer concentration [*A*_2_] with monomer concentration [*A*_1_] or [*A*_1_,*_tot_*] according to *AA* [10,32] or *AB* [42,43] mechanisms, as introduced in the previous section. The *AA* mechanism proceeds initially via *A*_1_–*A*_1_ collision to form the *MV* dimer, with a forward collision rate of *k*_11_ and an *MV* evaporation rate of *E*_2*MV*_. The *MV* dimer then reacts with base compounds at the collision rate $k_{21}^{\prime }$ to form an *LV* dimer, followed by collision with a monomer at collision rate *k*_21_. The dimer concentration [*A*_2_] can be derived as a function of the monomer concentration according to [10,32]:

(2) $$\left[A_{2}\right]=\frac{\frac{1}{2}k_{11}k_{21}^{\prime }\left[B\right]}{E_{2MV}+k_{21}^{\prime }\left[B\right]+\kappa }\left(\frac{{\left[A_{1}\right]}^{2}}{k_{21}[A_{1}]+\kappa }\right)\;$$

(2^′^) $$\left[A_{2}\right]=m\left(\frac{{\left[A_{1}\right]}^{2}}{k_{21}[A_{1}]+\kappa }\right)\;$$

where $m=\frac{\frac{1}{2}k_{11}k_{21}^{\prime }\left[B\right]}{E_{2MV}+k_{21}^{\prime }\left[B\right]+\kappa }$ and $\kappa =\frac{c}{4}A_{Fuchs}$, in which *c* is the mean thermal velocity of the sulfuric acid dimer, and $A_{Fuchs}$ is the Fuchs surface area of the aerosols, which can be obtained from the particle number size distribution of the SMPS. [*B*] is the concentration of the base compounds. 

Alternatively, we can also derive an analytical expression for [*A*_2_] as a function of [*A*_1_,*_tot_*] according to the *AB* mechanism [43]. Here, we keep the same notation for the rate constants as those in the SI of [43]. In polluted environments, the dimer concentration [*A*_2_] can be expressed as:

(3) $$\left[A_{2}\right]=\frac{k_{1}k_{3}\left(k_{2}+k_{10}\right)+k_{1}^{2}k_{4}\left[B\right]}{k_{12}{\left(k_{2}+k_{10}+k_{1}\left[B\right]\right)}^{2}}\left[B\right]{\left(\left[A_{1,tot}\right]\right)}^{2}\;$$

(3^′^) $$\left[A_{2}\right]=s{\left(\left[A_{1,tot}\right]\right)}^{2}\;$$

where $s=\frac{k_{1}k_{3}\left(k_{2}+k_{10}\right)+k_{1}^{2}k_{4}\left[B\right]}{k_{12}{\left(k_{2}+k_{10}+k_{1}\left[B\right]\right)}^{2}}\left[B\right]$. Note that the notation of monomer concentration used in Equations (2) and (3) is different due to the different initial steps of the cluster formation in the two mechanisms. In the *AB* mechanism, the sulfuric acid monomers react with a base compound to form *AB*, instead of with the monomer itself to form the *MV* dimer, leading to a loss of monomers to the base compound in the form of *A*_1_*B*_n_ (n = 1, 2, 3…). Here, we only consider the most important *AB* complex, *A*_1_*B*_1_, so that [*A*_1_,*tot*] = [*A*] + [*A*_1_*B*_1_]. The rate coefficients in Equation (3) are listed as follows, according to [43]: the collision rate between the monomer and the base, $k_{1}=\beta_{AB}$; the evaporation rate of *A*_1_*B*_1_, $k_{2}=\gamma_{1}$; the collision rate between *A*_1_*B*_1_ and the monomer,$\;k_{3}=\beta_{1A}$; the collision rate between the two *A*_1_*B*_1_ complex, $k_{4}=0.5\beta_{11}$; the condensation sink for *A*_1_*B*_1_, $k_{10}={\mathrm{CS}}_{1}$; and the condensation sink for *A*_2_*B*_1_ or *A*_2_*B*_2_, $k_{12}={\mathrm{CS}}_{2}$. A summary of the parameters used in Equations (2) and (3) is provided in the Supplementary Material (Table S2), including the values, or the range of values, and the methods used to calculate the parameters. 

## 3. Results and Discussion

Ten NPF events during the campaign were chosen for this study. *BGC* based on the above-mentioned *m/z* 166 protocol was performed for every event except the events on 6, 7, 10, and 12 August, and the fitting parameters are included in the Supplementary Material (Table S1). The correlations between sulfuric acid dimer concentration and monomer concentration were then investigated with the constraining amine concentrations during the events, according to the *AA* and *AB* theories. Evaporation rates of dimers and *AB* complexes were estimated based on the different acid–base mechanisms. Furthermore, in this section, the factors affecting the evaporation rates are discussed. 

### 3.1. Sulfuric Acid and Dimer Concentrations during NPF Events

**Figure 1:**
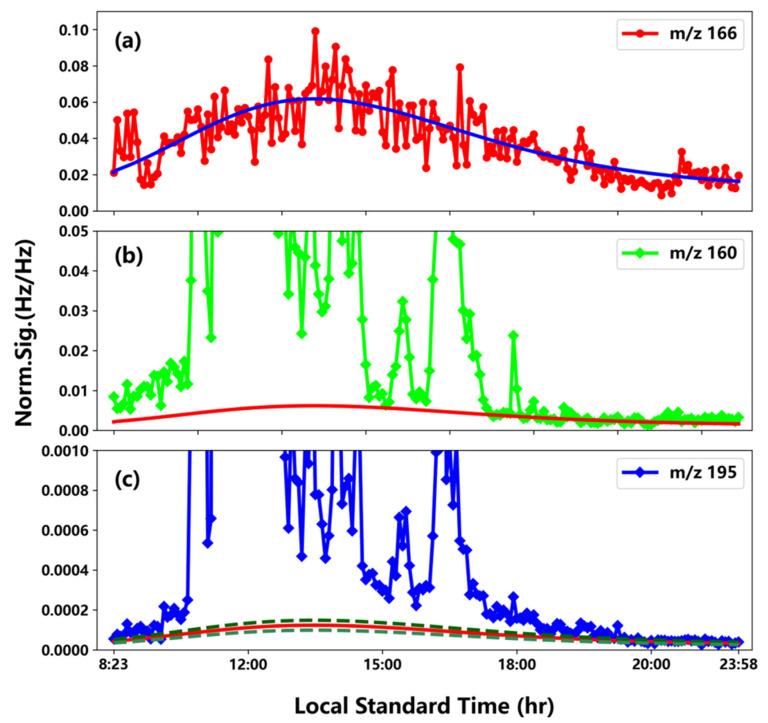
Temporal profiles of lognormal function fit for *m/z* 166 (**a**), and *BGCs* for *m/z* 160 (**b**) and *m/z* 195 (**c**), during the event on 22 August. See the main texts for details.

As an example of the *BGC* on the event of 22 August, Figure 1 shows the lognormal function fit for *m/z* 166 (a, blue), the scaled *BGCs* taken for *m/z* 160 with an *SF* of 0.0016 (b, red), and *m/z* 195 with an SF of 0.0667 (c, solid red), respectively. The dashed lines in the upper and lower sides of the solid red line correspond to ± 20% values from those of the red line. The fitting parameters *x*_0_, *y*_0_, *A*, and width are 48399, 0.00127, 0.0406, and 0.358, respectively. These fitting parameters, along with the *SFs* and fitting figures, are included in the Supplementary Material for other events, except 12 August, on which limited data were available on *BGC*, and 6, 7, 10 August, on which special processing was applied, as mentioned in the previous section. As shown in Figure 1, the lognormal function fits the normalized signal of *m/z* 166 very well, and an obvious smooth peak was seen at late noon (~14:00), implying its photochemical origin, and likely a temperature effect during the day. Figure 1 also strongly indicates that the BGs of both *m/z* 160 and 195 are far lower than their real signals during the event, and the *BGCs* contribute insignificantly to the total concentrations of sulfuric acid monomers and dimers. 

**Figure 2:**
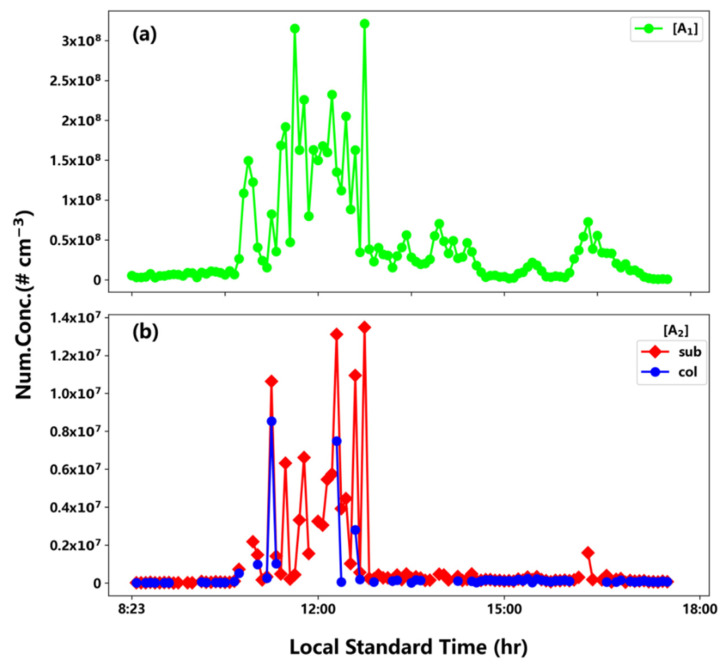
Temporal profiles of sulfuric acid concentration (**a**) and neutral dimer concentrations (**b**) with sub- or collision rate IIC corrections during the event on August 22.

The background-corrected sulfuric acid concentrations during the events were calculated according to the method used by Zhao et al. [9]. As mentioned in the previous section, the concentrations of neutral sulfuric acid dimers were obtained by subsequently subtracting background and the portion of ion-induced clustering (IIC). The rate coefficient for the IIC, as aforementioned, is not experimentally measured, and was empirically set to be 8 × 10^−10^ cm^3^ s^−1^ by fitting the measurement data of a previous study [9]. Here, we adopt this value as the sub-collision rate (sub-), and also the collision rate, for comparison. Note that the adopted sub-collision rate constant for IIC correction might lead to some uncertainties. Figure 2 shows temporal profiles for sulfuric acid concentrations (a) and neutral dimer concentrations with either sub- or collision rate corrections for the IIC corrections (b), during the event on 22 August. As shown in Figure 2, most of neutral dimer concentrations with the IIC collision rate correction were under the detection limit (UDL), implying that the rate coefficient of the IIC is indeed sub-collisional. Table 1 summarizes the average concentrations of sulfuric acid monomers and dimers with sub-collision and collision rate corrections for all the events during the campaign. The sulfuric acid monomer concentrations fall in the range of 1.7 × 10^7^–1.4 × 10^8^ cm^−3^, corresponding to neutral dimer concentrations of 4.1 × 10^5^–5.0 × 10^6^ cm^−3^ and 2.6 × 10^5^–2.7 × 10^6^ cm^−3^ for sub-collision and collision IIC corrections, respectively. The dimer and monomer concentrations are consistent with those reported at the same site in previous studies [10,25]. Variations in both sulfuric acid monomer and dimer concentrations were within an order of magnitude among all the events. Dimer concentrations with sub-collision correction were about 1.5 times those with collision correction. In addition, Table 1 and Figure 2 show that the dimer concentrations varied substantially with dramatic changes in monomer concentrations, and probably with amine concentrations as well, as will be explored more in Section 3.3. 

The apparent conversion ratio of sulfuric acid dimer to monomer signals (not IIC-corrected for the dimer signals) was also calculated, corresponding to the maximum ability of converting monomers to dimers. Table 2 shows the different percentage values of the conversion ratios for all the events of interest, along with the corresponding correlation coefficients (R^2^) and the event types. In general, the dimer signals (*m/z* 195) were well correlated with those of the monomers, with all correlation coefficients being over 0.7, and even as high as 0.93 for 7 August (regional and plume mixed type). The conversion ratios were in the range of 3.6–13.7 %, much lower than the upper limit calculated from the hard-spherical collision theory (18 %). Almost all the events, except the above-mentioned August 7 event, were plume-type, indicating the influence of the emissions from local coal-fired plants, which emit high amounts of sulfur dioxide into the atmosphere and lead to the abrupt increase of particle number concentrations during NPF events.

### 3.2. Evaporation Rates of Dimer or Sulfuric Acid–Base Complex

**Figure 3:**
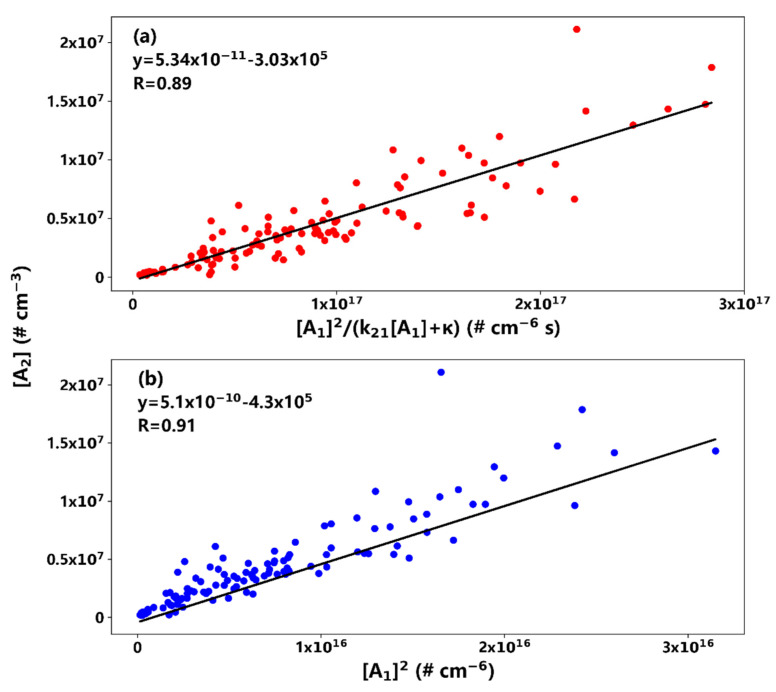
The dimer concentration [A_2_] plotted as a function of $\frac{{\left[A_{1}\right]}^{2}}{k_{21}[A_{1}]+\kappa }$ (**a**) and [A_1_]^2^ (**b**) during the NPF event on 12 August. The fitted function is shown in the figure, along with the Pearson correlation coefficient.

As mentioned in the introduction, two mechanisms were proposed in sulfur-rich environments such as Atlanta (*AA* vs. *AB*), and the dependence of dimer concentration [*A*_2_] on monomer concentration [*A*_1_] is different between *AA* and *AB* mechanisms (Equations (2) and (3)). In *AA*, [*A*_2_] is proportional to [*A*_1_] if the loss of the dimers to trimers is much larger than to the pre-existing particles; that is, $k_{21}[A_{1}]\gg \kappa$, or oppositely, if $k_{21}[A_{1}]\ll \kappa$, [*A*_2_] is proportional to [*A*_1_]^2^. In *AB*, [*A*_2_] is always proportional to [*A*_1_]^2^. During all of the NPF events in this study, the κ values vary insignificantly (Table 3), indicating a relatively constant condensational sink during the events. Figure 3a shows the linear regression between the dimer concentration [*A*_2_] and $\frac{{\left[A_{1}\right]}^{2}}{k_{21}[A_{1}]+\kappa }$ during the NPF event on 12 August, and a fitting coefficient of 5.34 × 10^−11^ cm^3^ s^−1^ was determined with a Pearson correlation coefficient of about 0.9. Since the fitting coefficient *m* is a function of both the evaporation rate *E*_2*MV*_ and the amine concentration [*B*], we calculated *E*_2*MV*_ based on average, minimum, and maximum [*B*]. In this case, the $k_{21}[A_{1}]/\kappa$ ratio is about 0.45, indicating that there is a significant condensational sink of the *LV* dimers compared to their loss to trimers. The calculated evaporation rates of the *MV* dimers range from 0.1–1.3 s^−1^ according to the *AA* mechanism, under average amine concentrations of about 22–48 ppt (Table S3). 

Similarly, Figure 3b shows the correlation between the dimer concentration [*A*_2_] and [*A*_1_]^2^ during the same event on 12 August, and a fitting coefficient of 5.10 × 10^−10^ cm^3^ molecule^−1^ was determined, with a similar Pearson correlation coefficient (0.9). In the *AB* mechanism, the fitting coefficient *s* is a function of both the evaporation rate of *AB* and the amine concentration [*B*] and, similar to the condensational sink of dimer, the two sink terms in *s* were also relatively constant. The evaporation rates of *AB* were calculated to be 1.2–13.1 s^−1^ according to the *AB* mechanism, based on the same range of average amine concentrations summarized in Table 4. Note that the *p* values for both *AA* and *AB* correlations were far lower than 0.01 (<10^−7^ for all cases except those on 22 and 23 August, as will be discussed below), indicating statistical importance for these correlations. The evaporation rates of *AB* via the *AB* mechanism were much larger than the corresponding values of the *MV* dimer via the *AA* mechanism, indicating that sulfuric acid dimer formation may likely be initiated through the *AA* mechanism in sulfur-rich polluted environments such as Atlanta, under several tens of parts per trillion by volume of amine concentration. However, since both the *AA* and *AB* mechanisms are overly simplified, and the amine concentrations may vary significantly during the events, more field measurements and laboratory experiments with better constrained conditions are needed to fully explore the initial step of atmospheric nucleation. 

**Figure 4:**
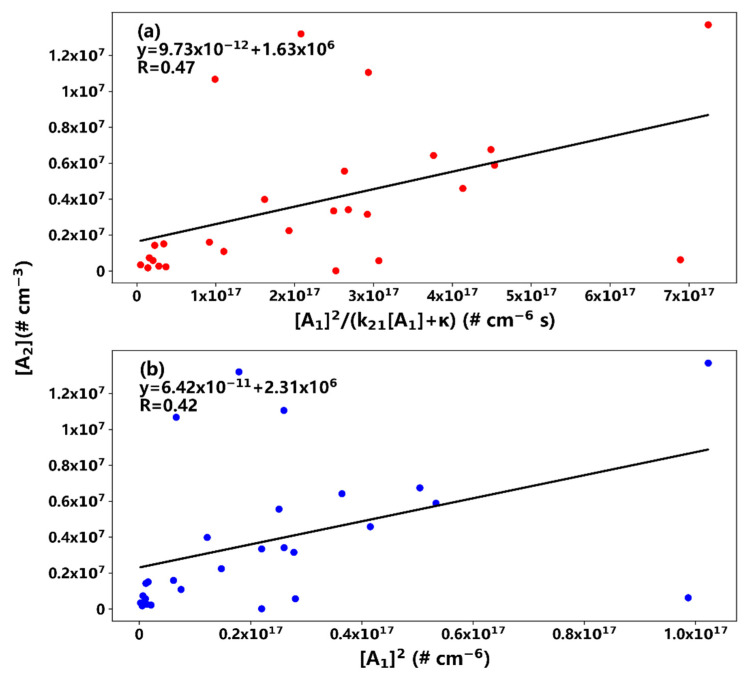
Same as for Figure 3, but for the event on 22 August.

It should be pointed out that for the NPF events on 22 and 23 August, poor correlations between dimer concentrations and $\frac{{\left[A_{1}\right]}^{2}}{k_{21}[A_{1}]+\kappa }$ (via the *AA* mechanism) and [*A*_1_]^2^ (via the *AB* mechanism) were found. As shown in Figure 4, the Pearson correlation coefficients of the above-mentioned relationships were only about 0.4, indicating that the mechanism responsible for the initial cluster formation during those two events might be significantly different from *AA* and *AB* mechanisms. The average concentrations of sulfuric acid monomers were about 1.3 × 10^8^ and 1.4 × 10^8^ cm^−3^, the two highest values among all the events in this study. The loss of dimers to trimers was significant compared to the condensation sinks in the *AA* mechanism, and the $k_{21}[A_{1}]/\kappa$ ratios were about 0.9 and 1.4 for the events on 22 and 23 August, respectively, much higher than values during other events. This implies that dimer formation might not be the bottleneck of the nucleation process, and the rate-determining step for the cluster formation might be trimer or higher cluster formation. The non-linearity of the correlations is likely attributed to the non-linearity of the kinetic and thermodynamic processes between the dimers and monomers of sulfuric acid. As shown in many previous laboratory experiments, the critical nucleating clusters might contain three or more sulfuric acid molecules under high monomer concentrations and/or low temperatures. Another notable feature during the above two events is the generally lower temperature; for example, the average temperature was 29 and 23 °C during the events on 22 and 23 August, respectively, compared to an average temperature of 32 °C during other events. Low temperatures also promote initial sulfuric acid dimer formation, which may contribute to the observed weak correlation shown in Figure 4.

### 3.3. The Effects of Amines on Sulfuric Acid Dimer Formation

Basic compounds, such as ammonia and amines, were previously shown to enhance atmospheric nucleation according to field and laboratory measurements. Here, we further explored the effects of the amines on sulfuric acid dimer formation, focusing primarily on the evaporation rates of *MV* dimers as we speculated in the previous section that the *AA* mechanism was more likely in sulfur-rich environments. Since no evaporation rates during the two events on 22 and 23 August were determined, we excluded these two events, thus only eight events in this study are discussed. The average amine concentrations were in the range of 22–48 pptv, and varied to some extent during the events. The main amine species were dimethyl amine (mw = 45), trimethyl amine (mw = 59), and triethyl amine (mw = 101), and their concentrations were dependent on temperature, especially for triethyl amine, which was the most abundant amine in this study. The temperature dependency of the amine concentrations might indicate the soil and vegetation origin of the amines [51]. 

**Figure 5:**
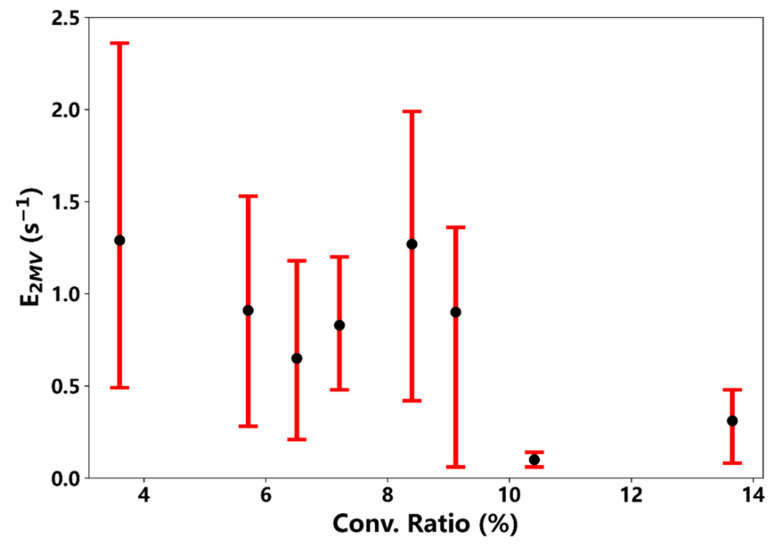
Plot of the evaporation rate of *MV* dimers vs. the apparent conversion ratio of monomers to dimers during the NPF events in this study (except those on 22 and 23 August). The black dots represent the averaged values and the error bars (red lines) represent the evaporation rates corresponding to maximum (upper) and minimum (lower) [*B*].

Figure 5 shows the relationship between the evaporation of dimers and the apparent conversion ratios of monomers to dimers. In general, as fewer dimers were evaporated, more monomers were converted to dimers, leading to more effective dimer formation during the events. Although the evaporation rate varied significantly during individual events, its deceasing trend with the conversion ratio was clearly shown. During a specific event, once the fitting coefficient was determined, the evaporation rate was linearly proportional to the amine concentration, as can be derived from the *m* formula (*m* in Equation (2)). In addition to the amine concentration, *m* was also affected by the coagulation scavenging parameter, κ. The average κ values ranged from 0.03 to 0.07 s^−1^, and as mentioned above, the loss to trimers ($k_{21}[A_{1}])$ was about 0.1 to 0.5 of the corresponding κ value (last column in Table 3), indicating that scavenging effects likely play a significant role in dimer formation. Subsequently, we used the total amine concentration to calculate the evaporation rates, assuming that the contribution of amine alone was equal and not discriminable. In addition, the average concentration of TMA or TEA was more than five times that of the DMA during the events in this study, indicating that these two amines may play important roles in stabilizing dimers. However, the effects of different amines might be different, and so this equally additive assumption might lead to large uncertainties in determining the effective concentrations of the total amines when calculating the evaporation rates. Hence, large uncertainties exist for the evaporation rates listed in both Table 3 and Table 4, and more work is needed to explore the relative contribution of individual amine to the evaporation rate. 

## 4. Conclusions

In sulfur-rich environments, previous field measurements have found that sulfuric acid dimer formation is the bottleneck of the initial step in atmospheric nucleation. In this study, sulfuric acid monomer and dimer concentrations measured by the Cluster CIMS were reported and employed to estimate the evaporation rates of sulfuric acid dimers and sulfuric acid–amine complexes under two acid–base nucleation mechanisms. 

A photochemical origin of signal *m/z* 166 was employed as a reference to correct the backgrounds of the monomer and dimer signals during new particle formation events through scaled lognormal functional fit. However, the background corrections were found to be minor compared to both sulfuric acid monomer and dimer signals during the events. The neutral sulfuric acid dimer concentrations were additionally corrected by ion-induced clustering (IIC) with either sub-collision rate (8 × 10^−10^ cm^3^ s^−1^) or collision rate (1.9 × 10^−9^ cm^3^ s^−1^). The sulfuric acid monomer concentrations were calculated to be in the range of 1.7 × 10^7^–1.4 × 10^8^ cm^−3^, and the corresponding neutral dimer concentrations were 4.1 × 10^5^–5.0 × 10^6^ cm^−3^ and 2.6 × 10^5^–2.7 × 10^6^ cm^−3^ for sub-collision IIC and collision IIC corrections, respectively. Unphysical negative values of dimer concentrations were obtained with the collision IIC correction, implying that the IIC was overcorrected with the collision rates. 

The evaporation rates of the sulfuric acid dimers and sulfuric acid–amine complexes were estimated through two different acid–base mechanisms (*AA* and *AB*) in sulfur-rich polluted environments. Based on the simultaneously measured average concentrations of total amines, the evaporation rates were calculated to be 0.1–1.3 s^−1^ for the dimers, much lower than those (1.2–13.1 s^−1^) for the acid–amine complexes. These lower evaporation rates for the dimers imply that atmospheric nucleation likely proceeds through the *AA* mechanism in the first step of atmospheric nucleation, that is, the formation of the more volatile dimers was more likely than sulfuric acid–amine complexes in sulfur-rich environments. The combined effects of sulfuric acid and amine concentrations, and the condensational sinks, for manipulating dimer formation were complicated, and the contribution of individual amine needs to be assessed in future studies in order to better understand the roles of amines in atmospheric new particle formation.

## Figures and Tables

**Table 1:** The average concentrations of sulfuric acid monomers and dimers with sub-collision rate corrections for all the events in this study.

Date	[*A*_1_] (cm^−3^)	[*A*_2_] (cm^−3^)	
		Sub-Col.	Col.
24 July	(4.09 ± 2.42) × 10^7^	(1.86 ± 1.55) × 10^6^	(1.22 ± 1.13) × 10^6^
25 July	(4.17 ± 2.47) × 10^7^	(2.09 ± 2.28) × 10^6^	(1.16 ± 1.60) × 10^6^
3 August	(6.22 ± 3.62) × 10^7^	(2.97 ± 3.20) × 10^6^	(1.66 ± 2.24) × 10^6^
6 August	(5.23 ± 4.09) × 10^7^	(1.81 ± 2.30) × 10^6^	(6.81 ± 7.30) × 10^5^
7 August	(3.03 ± 2.70) × 10^7^	(9.61 ± 9.91) × 10^5^	(4.94 ± 4.23) × 10^5^
10 August	(1.74 ± 1.60) × 10^7^	(4.08 ± 4.77) × 10^5^	(2.59 ± 3.14) × 10^5^
11 August	(5.74 ± 4.11) × 10^7^	(1.63 ± 1.72) × 10^6^	(5.62 ± 7.38) × 10^5^
12 August	(8.10 ± 3.87) × 10^7^	(5.01 ± 3.86) × 10^6^	(2.66 ± 2.23) × 10^6^
22 August	(1.29 ± 0.81) × 10^8^	(3.82 ± 4.04) × 10^6^	(2.28 ± 3.06) × 10^6^
23 August	(1.40 ± 0.76) × 10^8^	(2.04 ± 2.32) × 10^6^	(1.12 ± 1.47) × 10^6^

The errors were estimated based on the measured data during the events.

**Table 2:** The apparent conversion ratios and the correlation coefficients for all the events of the study, along with the types of NPF.

Date	Conversion Ratio (%)	Correlation Coefficient (R^2^)	NPF Type
24 July	7.21	0.76	Plume
25 July	10.41	0.83	Plume
3 August	9.12	0.72	Plume
6 August	8.40	0.85	Plume
7 August	5.71	0.93	Regional/plume
10 August	3.60	0.79	Plume
11 August	6.51	0.86	Plume
12 August	13.66	0.88	Plume
22 August	9.05	0.78	Plume
23 August	6.84	0.73	Plume

**Table 3:** The evaporation rates of the more volatile (*MV*) dimers based on the fitting coefficient (m) in Equation (2) for the *AA* mechanism, along with the correlation coefficient (Pearson) and other parameters.

Date	m (cm^3^ s^−1^)	Pearson Coeff. (R)	*κ* (s^−1^)	*E*_2*MV*_ (s^−1^) *			*k*_21_[*A*_1_]/*κ*
24 July	2.71 × 10^−11^	0.69	0.033 ± 0.006	0.83	0.48	1.20	0.37
25 July	7.80 × 10^−11^	0.90	0.052 ± 0.011	0.10	0.06	0.14	0.22
3 August	3.58 × 10^−11^	0.73	0.039 ± 0.007	0.90	0.06	1.36	0.47
6 August	3.17 × 10^−11^	0.91	0.032 ± 0.013	1.27	0.42	1.99	0.45
7 August	3.58 × 10^−11^	0.86	0.069 ± 0.031	0.91	0.28	1.53	0.17
10 August	2.56 × 10^−11^	0.76	0.037 ± 0.007	1.29	0.49	2.36	0.13
11 August	3.31 × 10^−11^	0.85	0.061 ± 0.024	0.65	0.21	1.18	0.25
12 August	5.34 × 10^−11^	0.89	0.049 ± 0.012	0.31	0.08	0.48	0.45
22 August	-	0.47	0.042 ± 0.004	-	-	-	0.89
23 August	-	0.48	0.029 ± 0.005	-	-	-	1.37

* The three columns for *E*_2*MV*_ correspond to average, minimum, and maximum [B] used in calculating the evaporation rates of the *MV* dimers.

**Table 4:** The evaporation rates of *AB* based on the fitting coefficient(s) in Equation (3) for the *AB* mechanism, along with the correlation coefficient (Pearson) and other parameters.

Date	s (cm^3^ molecule^−1^)	Corr. Coeff. (R^2^)	CS_1_ (s^−1^)	CS_2_ (s^−1^)	*k*_2_ (s^−1^) *		
24 July	4.49 × 10^−10^	0.71	0.036	0.025	4.76	2.7	6.77
25 July	8.17 × 10^−10^	0.91	0.056	0.040	1.17	0.94	1.46
3 August	4.27 × 10^−10^	0.73	0.042	0.030	6.44	3.8	9.69
6 August	3.14 × 10^−10^	0.92	0.035	0.024	13.12	4.48	20.3
7 August	3.07 × 10^−10^	0.89	0.076	0.539	5.07	1.75	8.29
10 August	4.11 × 10^−10^	0.78	0.041	0.029	6.49	2.58	11.8
11 August	2.7 × 10^−10^	0.86	0.067	0.047	4.41	1.64	7.71
12 August	5.44 × 10^−10^	0.91	0.053	0.037	2.55	0.86	3.81
22 August	-	0.42	0.045	0.032	-	-	-
23 August	-	0.44	0.032	0.023	-	-	-

* The three columns for *k*_2_ correspond to average, minimum, and maximum [*B*] used in calculating the evaporation rates of *AB*.

## Data Availability

All data are available at figshare (https://doi.org/10.6084/m9.figshare.19688932.v1, accessed on 5 February 2022).

## Supplementary Materials

The following supporting information can be downloaded at: https://www.mdpi.com/article/10.3390/ijerph19116848/s1, containing 2 text sections, 2 tables, and 8 figures. References [53,54] are cited in the supplementary materials.
