# Supplementary material for: Atmospheric Sulfuric Acid Dimer Formation in a Polluted Environment
Source: Int J Environ Res Public Health. 2022 Jun 3;19(11):6848. doi: 10.3390/ijerph19116848 (PMC9180914; doi:10.3390/ijerph19116848)
Supplement: Supplementary file 1 [file ijerph-19-06848-s001.zip › ijerph-1736633-SI.pdf]

Supplementary materials for

Atmospheric Sulfuric Acid Dimer Formation in a Polluted Environment

Ke Yin [1], Shixin Mai [1] and Jun Zhao [1, 2, 3, 4*]

[1] School of Atmospheric Sciences, Guangdong Province Key Laboratory for Climate Change and Natural Disaster Studies, and Southern Marine Science and Engineering Guangdong Laboratory (Zhuhai), Sun Yat-sen University, Zhuhai, Guangdong 519082, China
[2] Guangdong Provincial Observation and Research Station for Climate Environment and Air Quality Change in the Pearl River Estuary, Zhuhai, Guangdong 519082, China
[3] Key Laboratory of Tropical Atmosphere-Ocean System, Ministry of Education, Zhuhai, Guangdong 519082, China
[4] Guangdong Province Engineering Laboratory for Air Pollution Control, Guangdong Provincial Key Laboratory of Water and Air Pollution Control, South China Institute of Environmental Sciences, Ministry of Ecology and Environment, Guangzhou 510655, China

* Correspondence: zhaojun23@mail.sysu.edu.cn

This supplement contains 2 text sections, 2 tables, and 8 figures.

Text S1. The parameters used in Eq. (2).

In Eq. (2), the loss term $\kappa = \frac{c}{4} A_{Fuchs}$, c is the mean thermal velocity of the sulfuric acid dimer so that $c = \sqrt{\frac{8RT}{\pi M}} = \sqrt{\frac{8\times 8.314\times 303}{\pi\times 196\times 10^{-3}}} = 181\ m\ s^{-1}$

Here, T is the temperature that is set to about 303 K, the average temperature (300~307 K) during NPF events, except for the one on August 23 during which the temperature ranged from 291 to 302 K, resulting in a c value of 179 m s^{-1}. A_{Fuchs} is the Fuchs surface area of the aerosols and is calculated from the particle number size distribution (PNSD) of the SMPS. During the campaign, we only measure particle sizes from 3 nm up to about 500 nm and the Fuchs surface area A_{Fuchs} is calculated according to the PNSD in this size range. It ranges from xxx to xxx during NPF events selected for this study.

The forward rate constant, k_{11}, is taken to be hard sphere collision rate for sulfuric acid monomer using its bulk liquid density and k_{11} is approximately 4x10^{-10} cm^3 s^{-1} [1,2]. Similarly, we also approximate other collision rates (k'_{21} and k_{21}) to be ~4x10^{-10} cm^3 s^{-1}.

Text S2. The parameters used in Eq. (3).

The collision rates (β_{AB}, β_{1A}, β_{11}) between sulfuric acid monomer (A) and a base (B), between A_1B_1 and B, between A_1B_1 and itself, are also assumed to be hard sphere and are taken to be ~4x10^{-10} cm^3 s^{-1} so that $k_1 = \beta_{AB}$ =4x10^{-10} cm^3 s^{-1}, $k_3 = \beta_{1A}$ =4x10^{-10} cm^3 s^{-1}, $k_4 = 0.5\beta_{11}$ =2x10^{-10} cm^3 s^{-1}. The condensation sinks of A_1B_1 (k_{10}) and A_2B_1 or A_2B_2 (k_{12}) to the particles are assumed to be the same, that is, CS_1=CS_2.

Table S1. Fitting methods and parameters, along with the scaling factors used in background corrections of m/z 160 (monomer) and m/z 195 (dimer) signals.

Date	Fitting parameters				Scaling factors (SF)	
	y_0	A	x_0	width	m/z160	m/z195
**Lognormal function fitting** $y = SF * \left\{ y_0 + A \cdot \exp\left[-\left(\frac{ln\left(\frac{x}{x_0}\right)}{width} \right)^2 \right] \right\}$						
**0724**	0.001745	0.19084	54899	0.58544	0.025	0.0025
**0725**	-0.025076	0.18882	47054	0.54531	0.02	0.0013
**0803**	0.0099424	0.14233	48708	0.44401	0.01	0.001
**0811**	0.0025846	0.040628	49136	0.46895	0.05	0.0016
**0822**	0.012719	0.049058	48399	0.35803	0.0667	0.002
**0823**	-0.001533	0.0083448	53511	0.40023	0.001667	0.00142
**Other background correction methods**						
**0806**	$y=-2x10^{-5}x^2 + 0.0015x + 0.0263$				0.008	0.002
**0807**	$y=-2x10^{-5}x^2 + 0.0025x + 0.0407$				0.066	0.002
**0810**	BGs considered invariably during the day and set y=0.02				0.05	0.00105
**0812**	BGs uncorrected					

Table S2. Parameters and their corresponding values used in Eqs. (2) and (3).

Parameters	Value	Unit
$\mathbf{k}'_{21}$	4 x 10^{-10}	cm^3 s^{-1} $molecule^{-1}$
$\boldsymbol{k_{21}}$	4 x 10^{-10}	cm^3 s^{-1} $molecule^{-1}$
$\boldsymbol{k_{11}}$	4 x 10^{-10}	cm^3 s^{-1} $molecule^{-1}$
$\boldsymbol{k_1}$	4 x 10^{-10}	cm^3 s^{-1} $molecule^{-1}$
$\boldsymbol{k_3}$	4 x 10^{-10}	cm^3 s^{-1} $molecule^{-1}$
$\boldsymbol{k_4}$	2 x 10^{-10}	cm^3 s^{-1} $molecule^{-1}$
$\boldsymbol{c_{A_2}}$	181.5	m s^{-1}
$\boldsymbol{c_{AB}}$*	197.2	m s^{-1}
$\boldsymbol{c_{A_2B_2}}$*	139.3	m s^{-1}

* The molecular weight of B was averaged over those of dimethyl amine (45), trimethyl amine (59), and triethyl amine (101) when calculating the mean thermal velocity.

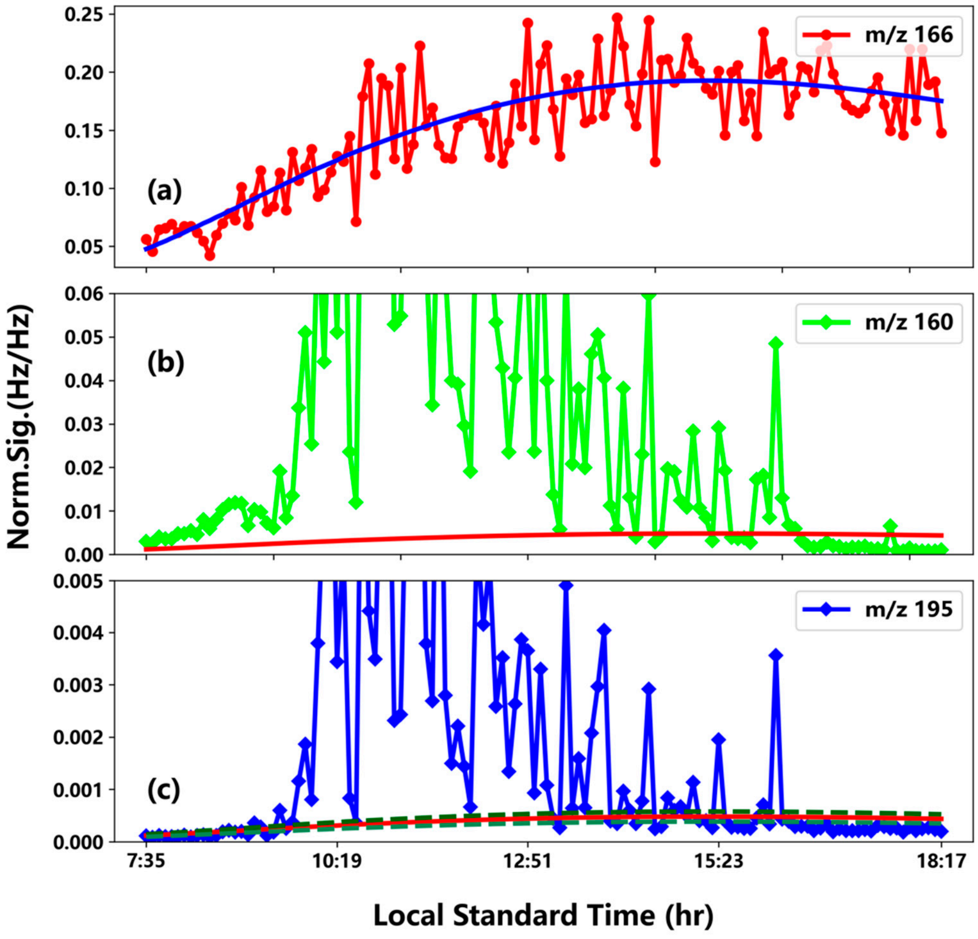

Figure S1. Lognormal function fit for BGC during the event on July 24.

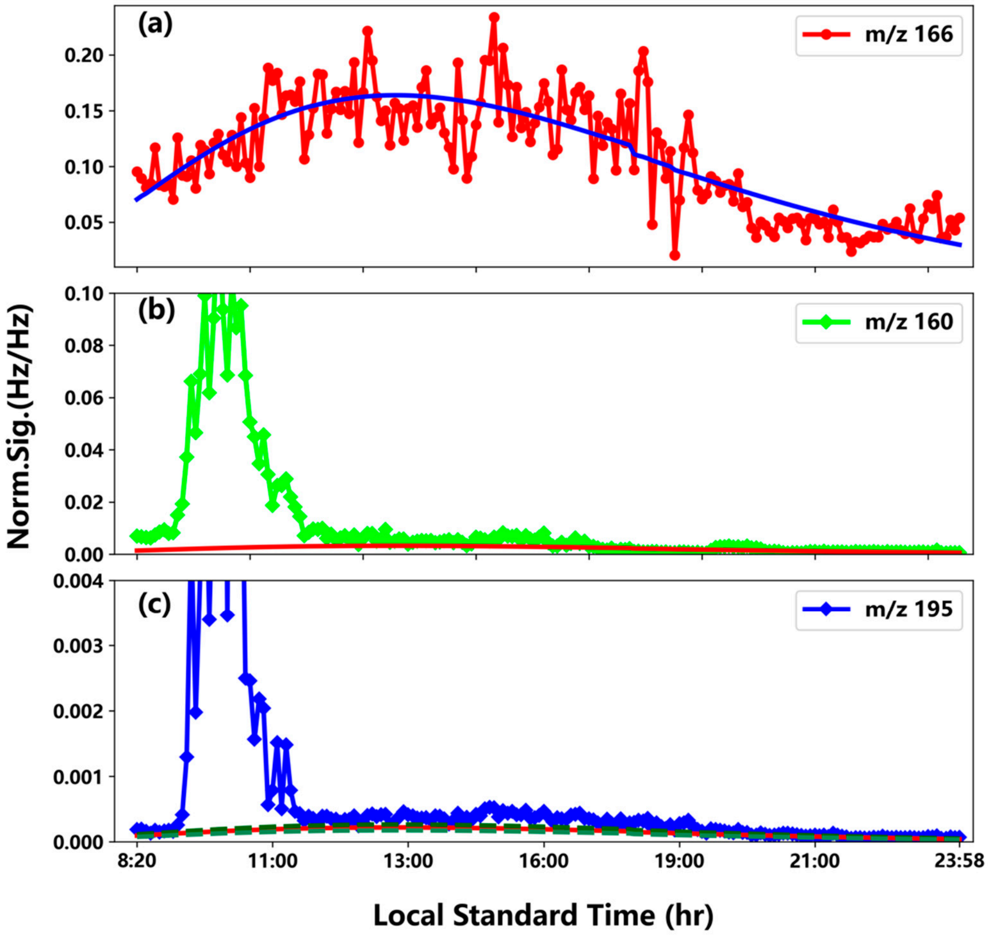

Figure S2. Lognormal function fit for BGC during the event on July 25.

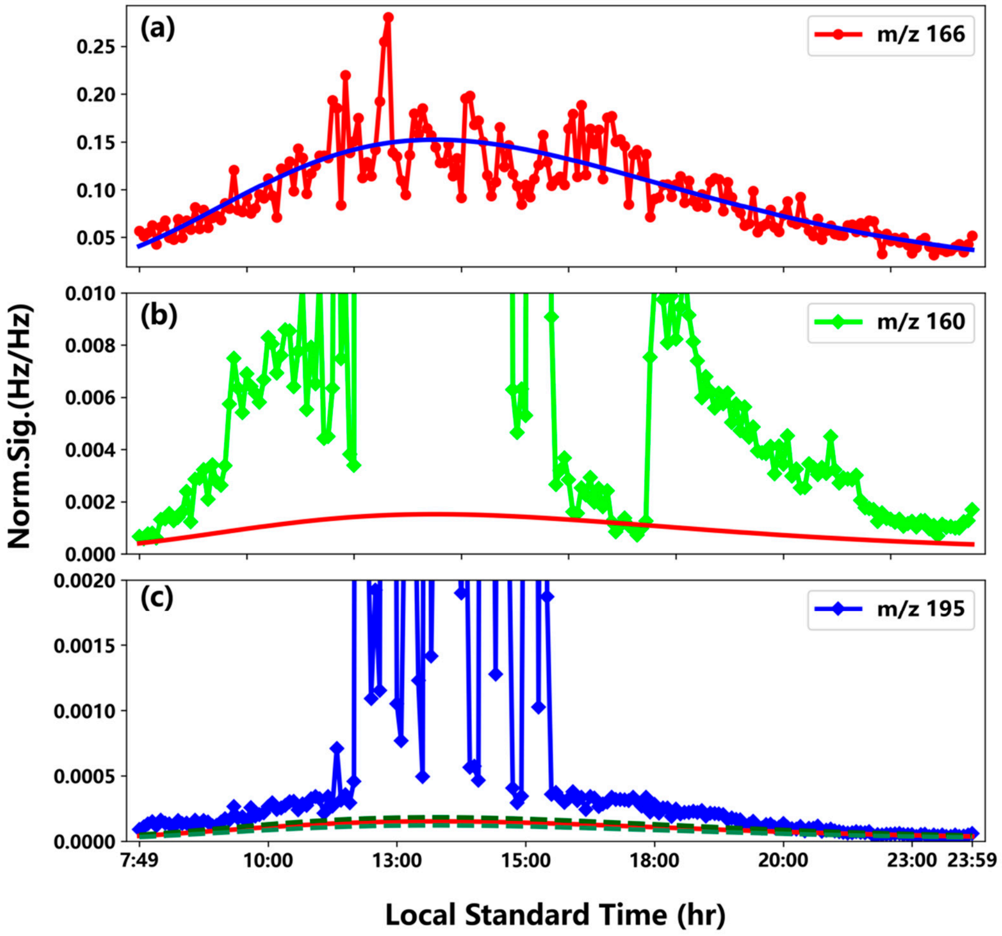

Figure S3. Lognormal function fit for BGC during the event on August 03.

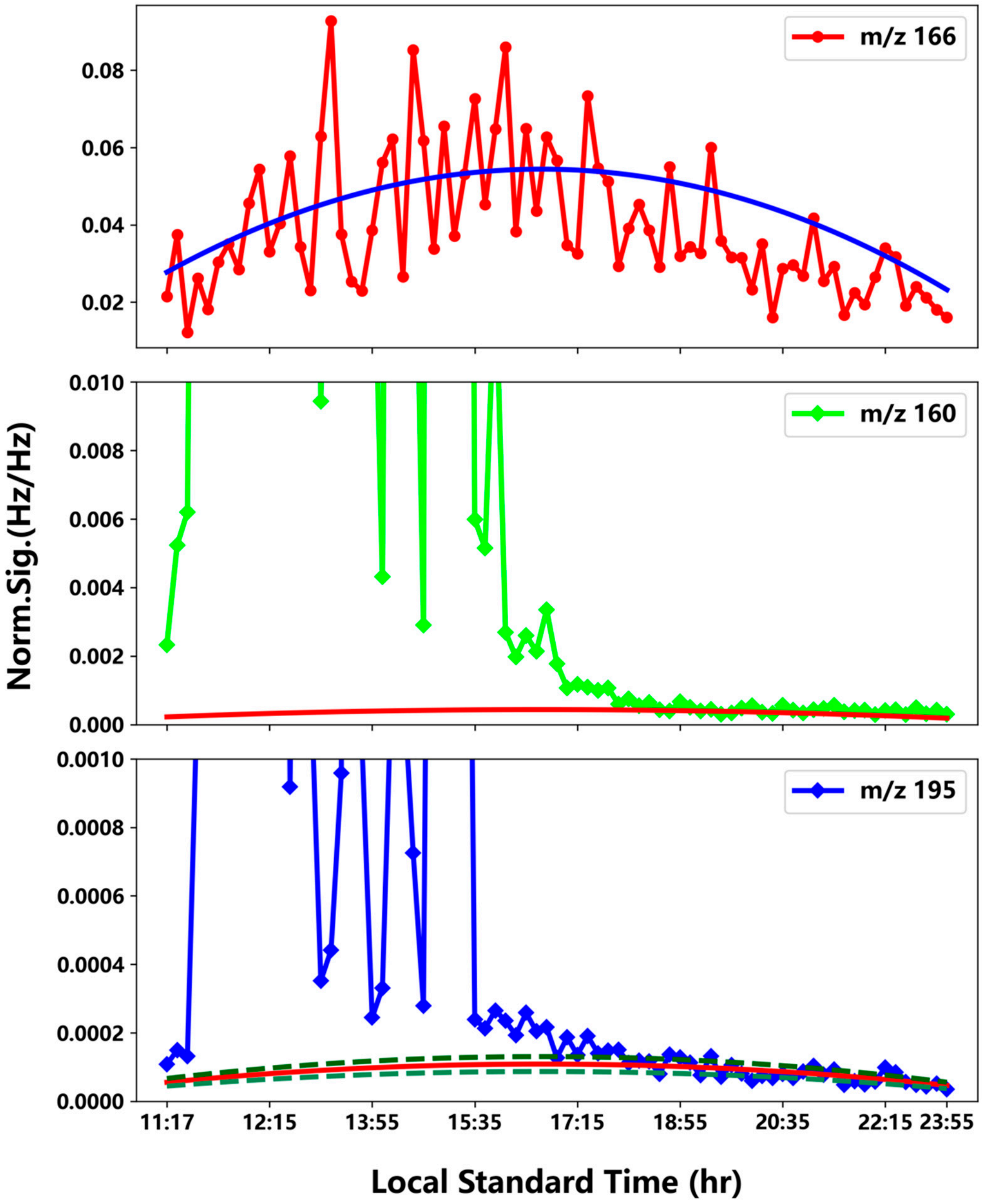

Figure S4. Quadratic function fit for BGC during the event on August 06.

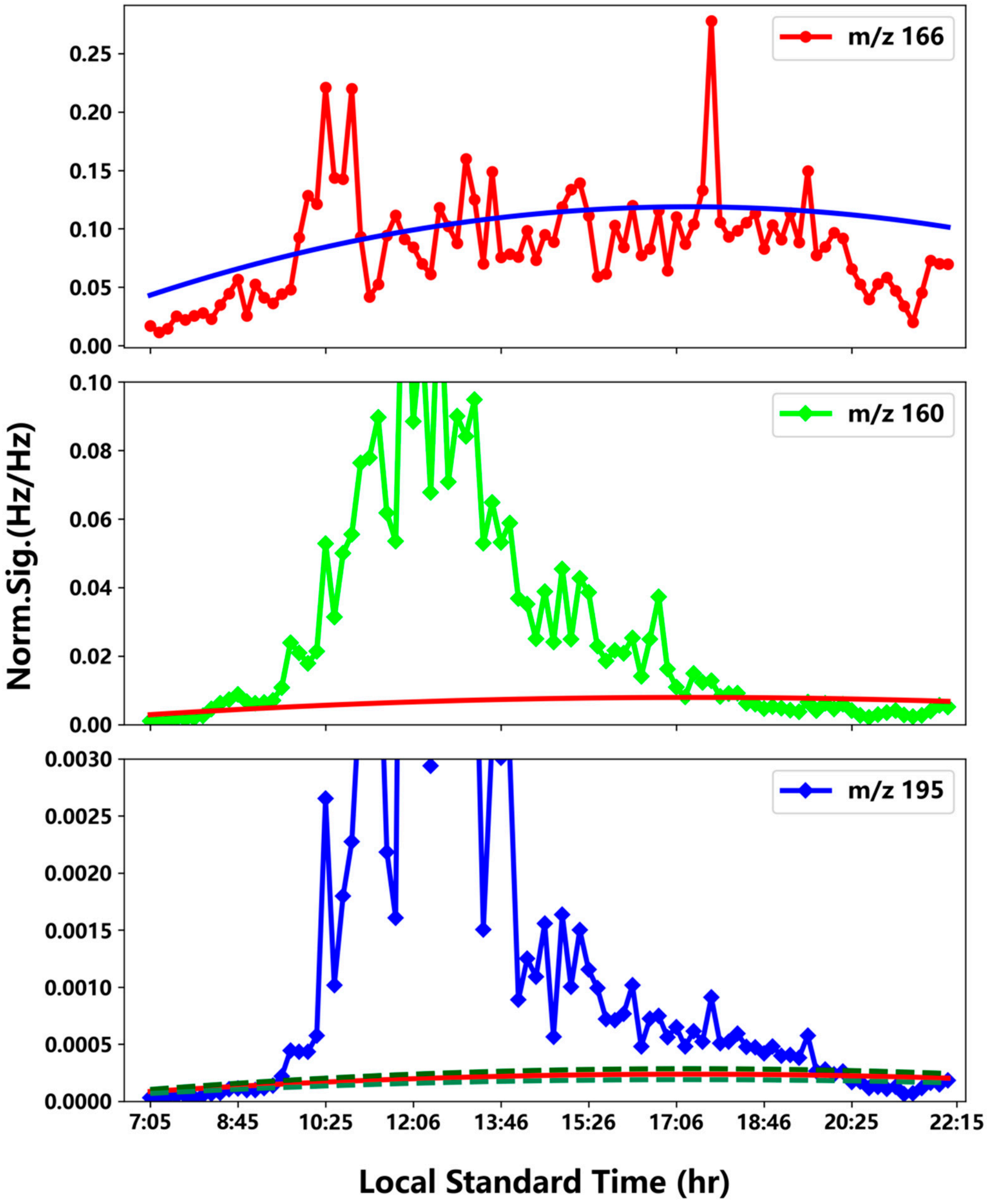

Figure S5. Quadratic function fit for BGC during the event on August 07.

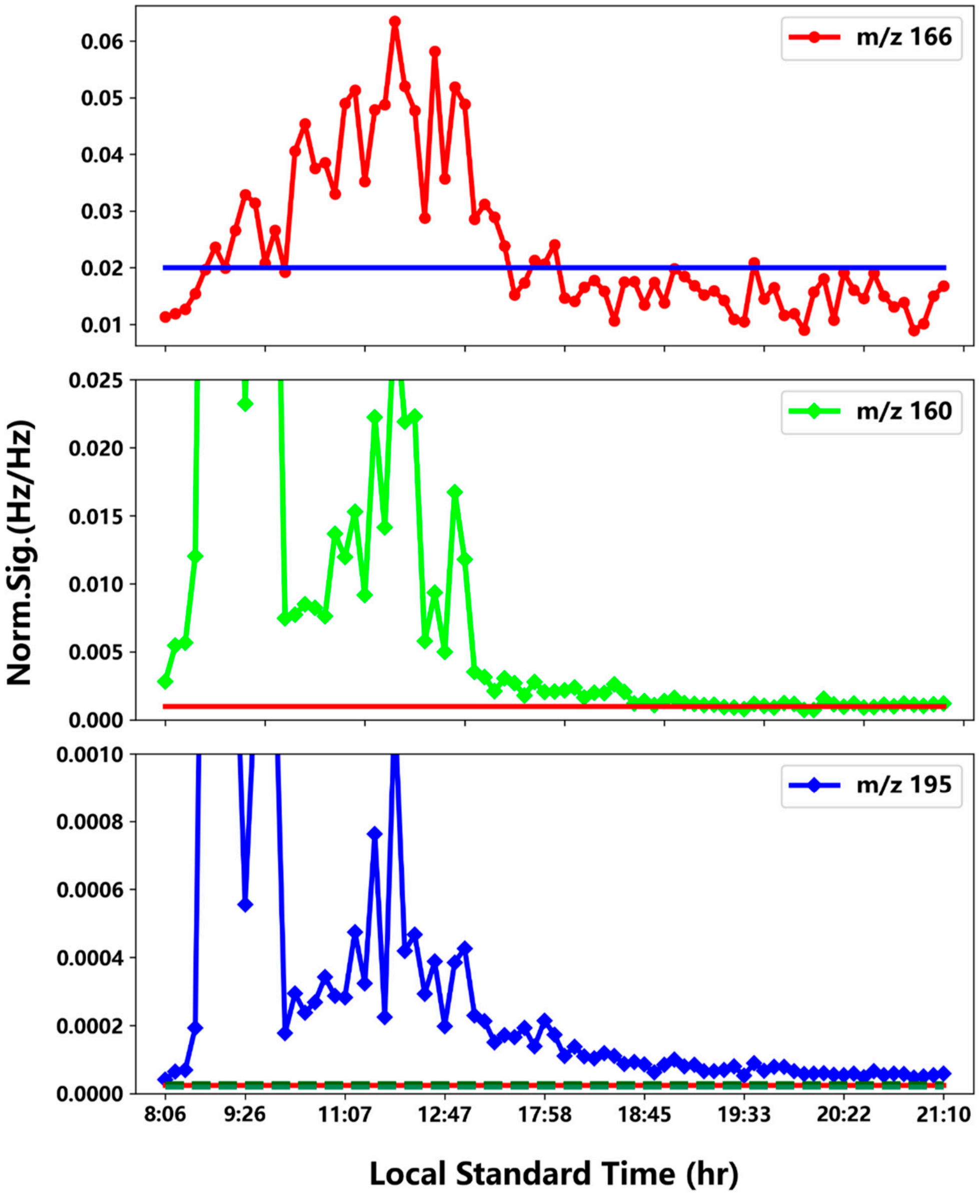

Figure S6. Almost no variation for backgrounds during the event on August 10.

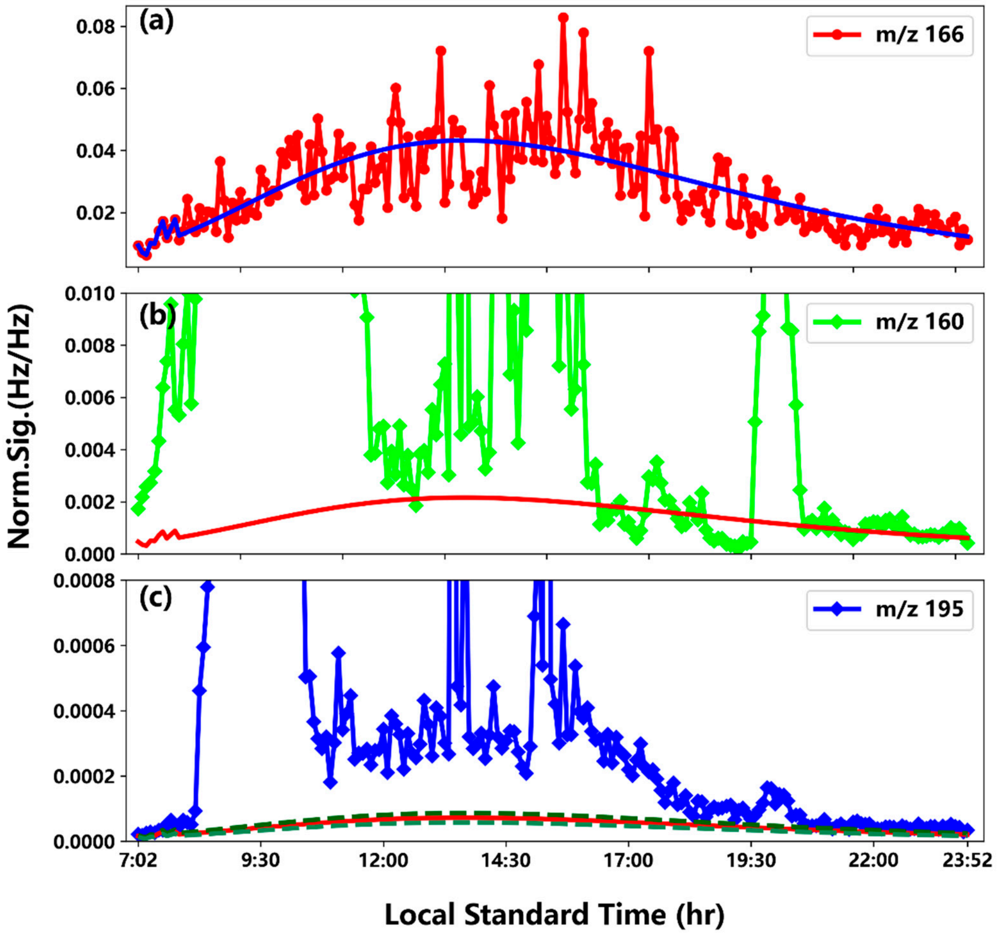

Figure S7. Lognormal function fit for BGC during the event on August 11.

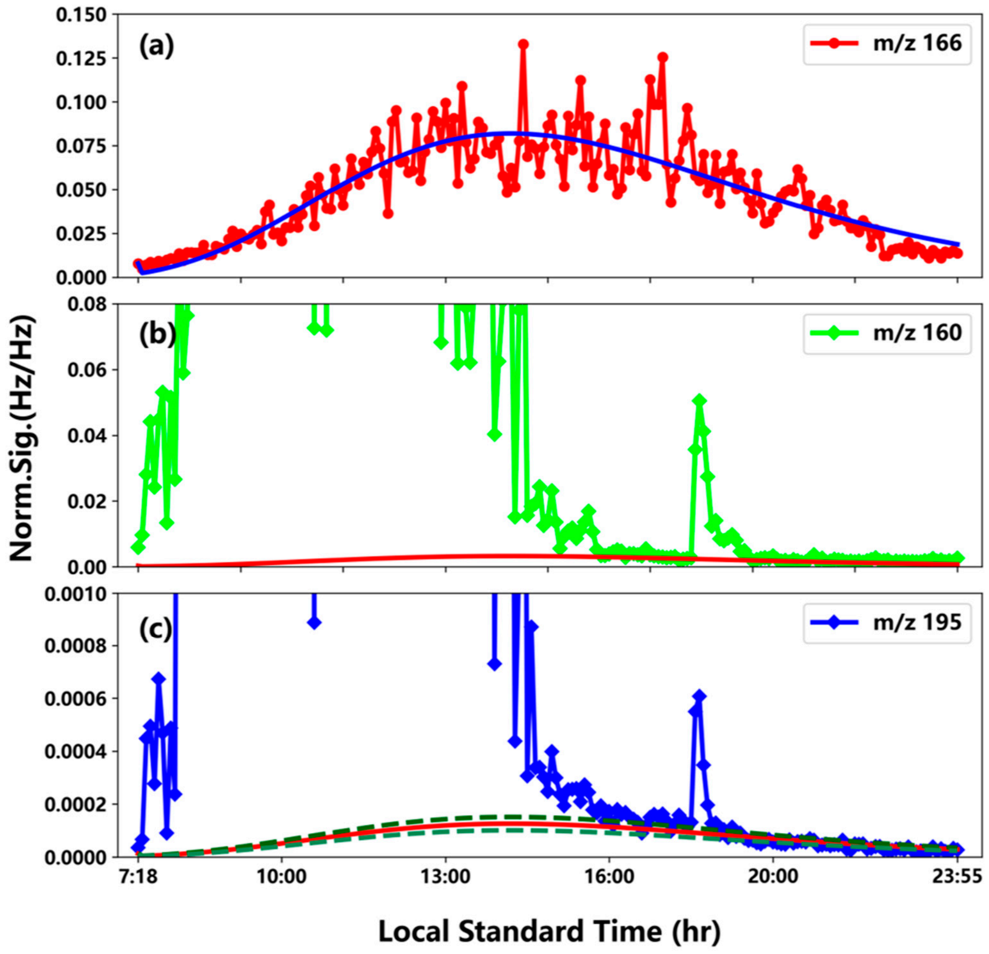

Figure S8. Lognormal function fit for BGC during the event on August 23.

References

1. McMurry, P H, 1983. New particle formation in the presence of an aerosol: Rates, time scales, and sub-0.01 μm size distributions. Journal of Colloid and Interface Science 95(1): 72-80. doi: http://dx.doi.org/10.1016/0021-9797(83)90073-5.
2. Ortega, I K, Kupiainen, O, Kurtén, T, et al., 2012. From quantum chemical formation free energies to evaporation rates. Atmospheric Chemistry and Physics 12: 225-235. doi:10.5194/acp-12-225-2012.
